# Effect of Hydroxyapatite Nanoparticle Crystallinity and Colloidal Stability on Cytotoxicity

**DOI:** 10.1021/acsbiomaterials.4c01283

**Published:** 2024-10-07

**Authors:** Lea Andrée, Lucas S. Joziasse, Merel J. W. Adjobo-Hermans, Fang Yang, Rong Wang, Sander C. G. Leeuwenburgh

**Affiliations:** † Department of Dentistry−Regenerative Biomaterials, Radboud University Medical Center, Nijmegen 6525 EX, The Netherlands; ‡ Department of Medical BioSciences, Radboud University Medical Center, Nijmegen 6525 GA, The Netherlands

**Keywords:** hydroxyapatite nanoparticles, crystallinity, agglomeration, cytotoxicity, intracellular calcium, reactive oxygen species

## Abstract

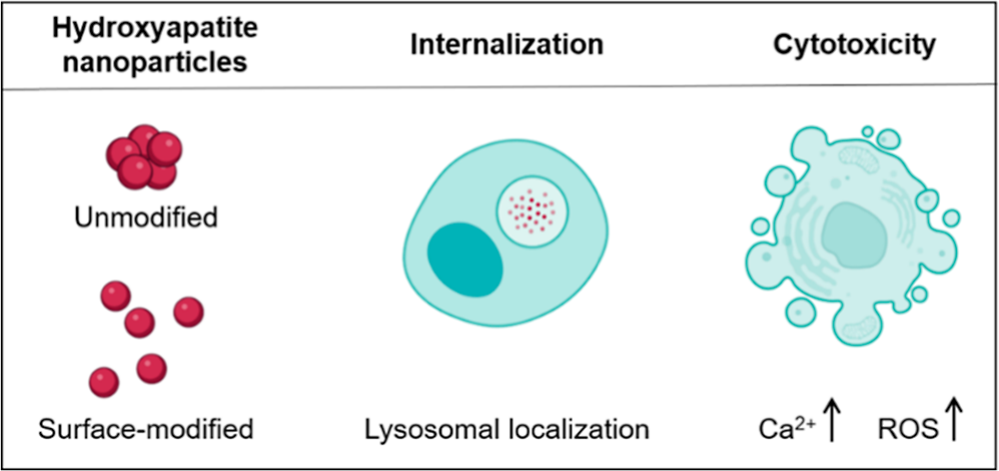

Hydroxyapatite nanoparticles (nHA) have gained attention as potential intracellular drug delivery vehicles due to their high binding affinity for various biomolecules and pH-dependent solubility. Yet, the dependence of nHA cytocompatibility on their physicochemical properties remains unclear since numerous studies have revealed starkly contrasting results. These discrepancies may be attributed to differences in size, shape, crystallinity, and aggregation state of nHA, which complicates fundamental understanding of the factors driving nHA cytotoxicity. Here, we hypothesize that nHA cytotoxicity is primarily driven by intracellular calcium levels following the internalization of nHA nanoparticles. By investigating the cytotoxicity of spherical nHA with different crystallinity and dispersity, we find that both lower crystallinity and increased agglomeration of nHA raise cytotoxicity, with nanoparticle agglomeration being the more dominant factor. We show that the internalization of nHA enhances intracellular calcium levels and increases the production of reactive oxygen species (ROS). However, only subtle changes in intracellular calcium are observed, and their physiological relevance remains to be confirmed. In conclusion, we show that nHA agglomeration enhances ROS production and the associated cytotoxicity. These findings provide important guidelines for the future design of nHA-containing formulations for biomedical applications, implying that nHA crystallinity and especially agglomeration should be carefully controlled to optimize biocompatibility and therapeutic efficacy.

## Introduction

Hydroxyapatite is widely used as a bone substitute material due to its compositional similarity to bone mineral, its ability to bind to host bone, and its osteoconductive activity. Hydroxyapatite has long been used in bulk or paste form in the orthopedic and dental fields, and shows favorable cyto- and biocompatibility characterized by low toxicity values.^1,2^ Lately, hydroxyapatite nanoparticles (nHA) have gained interest for other medical applications including bioimaging, cancer therapy, and drug delivery, due to their (i) high binding affinity to proteins and nucleic acids, (ii) easy surface modification for targeting, (iii) internalization by various cell types, (iv) faster degradation compared to other inorganic materials, and (v) pH-dependent solubility.^2,3^ Consequently, nHA is intensively studied to facilitate (intracellular) drug delivery. To this end, efficient cellular uptake and subsequent cargo release without causing cytotoxicity are crucial requirements for successful intracellular delivery.^4,5^

Although nHA is generally considered to be biocompatible and safe, in vitro cytotoxicity studies have reported contradicting results. While some studies indicate good cytocompatibility (>70% compared to control) even at concentrations of 300 μg/mL,^6−8^ others observed cytotoxicity already at concentrations below 10 μg/mL.^9,10^ Various other studies report cytotoxicity values between these extremes. These discrepancies may be attributed to differences in physicochemical properties of the tested nHA, alongside differences in the cell types and analytical methods used in different studies. In the biomedical field, nHA are generally characterized by their small size (typically several hundreds of nanometers) and corresponding high surface-to-volume ratio, resulting in high surface reactivity. Parameters such as size, shape, aspect ratio, surface characteristics, crystallinity, and aggregation state are known to affect cytocompatibility, but the primary determinants of nHA cytotoxicity remain unclear. For nHA, lysosomal rupture triggered by increased calcium concentration in the lysosome and/or generation of reactive oxygen species (ROS) are proposed,^11−13^ but the underlying mechanism of nanoparticle cytotoxicity is still not fully understood.

Several studies have investigated the effect of nHA shape on cytotoxicity, which revealed differences between different shapes (sphere > needle > rod), which correlates to an increase in specific surface area.^6,14,15^ Yet, the shape also determines the aspect ratio of nanoparticles, which in turn affects cellular uptake and dissolution rates. Similarly, nanoparticle dissolution rates increase with reducing particle size and thus increasing surface-to-volume ratio.^2,6,15^ A literature review suggests that nHA are not inherently toxic, and cytotoxicity is mostly observed for nonfunctionalized nanoparticles that tend to agglomerate.^16^ Yet, changes in intracellular calcium and cell death were reported for calcium phosphate-based nanoparticles.^7,8,10,17^ Based on the current state of the art, we therefore propose that observed differences in nHA cytotoxicity are ultimately related to nHA dissolution rate and hypothesize that nHA cytotoxicity is mainly dictated by the amount and rate of intracellular calcium release.

To test our hypothesis that intracellular calcium release is governing nHA cytotoxicity, we synthesized nHA with different crystallinity and colloidal stability (i.e., agglomeration) since both parameters are known to strongly affect nHA dissolution rate.^2^ First, we tested two approaches to vary crystallinity and colloidal stability of nHA by altering aging time and temperature during synthesis: surface modification using citrate as dispersant or lyophilization. After in-depth physicochemical characterization and cytotoxicity assessment of these nHA, we selected two nHA nanoparticles with different degrees of agglomeration to study cellular internalization, intracellular calcium, and ROS production in more detail.

## Results and Discussion

### Physicochemical Properties and Cytotoxicity of Hydroxyapatite Nanoparticles as a Function of Citrate Modification and Aging Time

**Figure 1:**
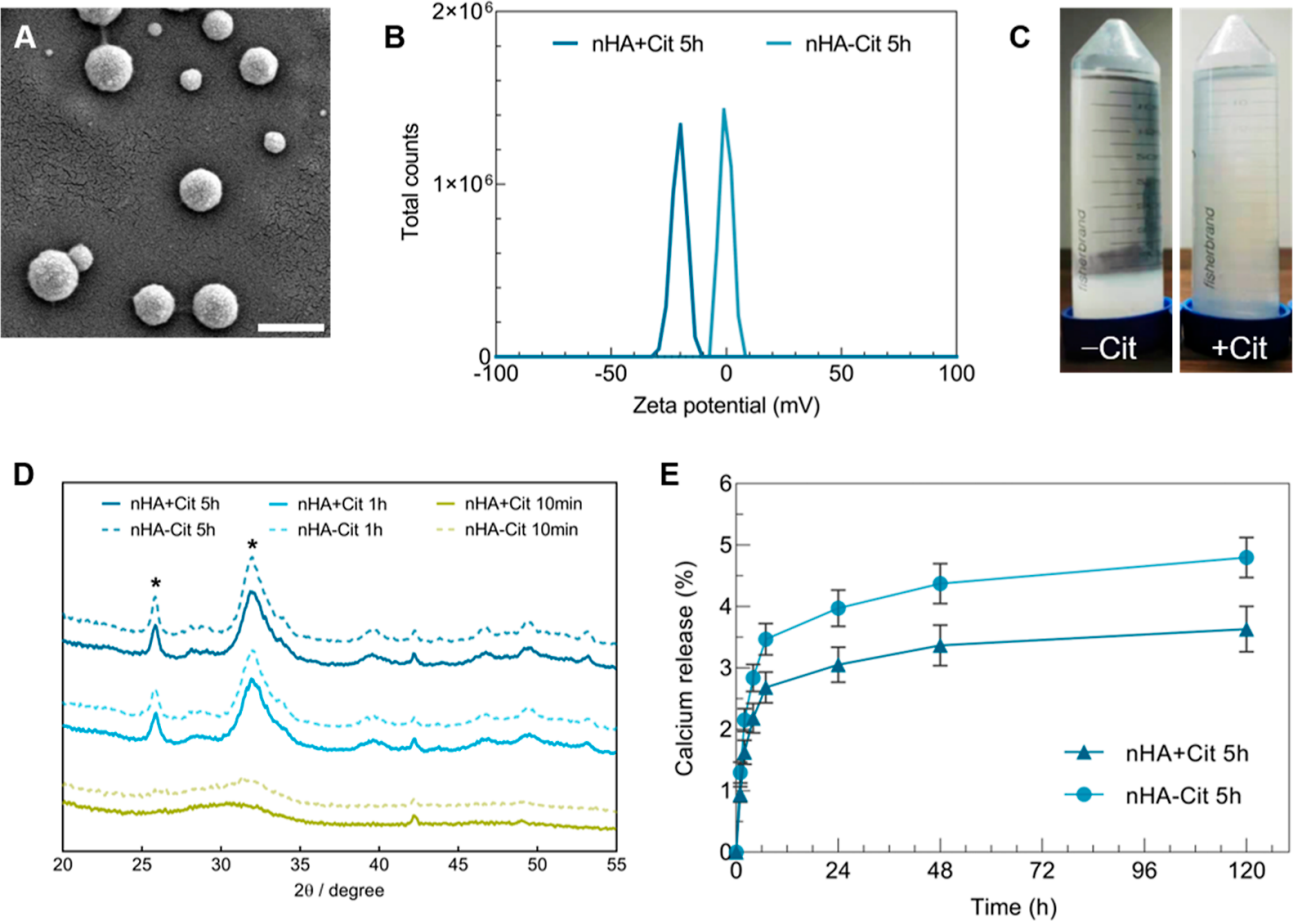
Physicochemical characterization of nHA. (A) Scanning electron microscopy image of nanoparticle morphology, (B) zeta potential distribution, and (C) photographs of suspensions of nHA synthesized with (+)/without (−) citrate as dispersant. (D) X-ray diffractograms of nHA as a function of citrate addition (with (+)/without (−) citrate) and aging time. (E) Calcium release at pH 6. All nanoparticles were synthesized at 40 °C. * indicates peaks characteristic for apatite. Scale bar represents 500 nm.

Hydroxyapatite nanoparticles (nHA) were first synthesized by a wet chemical one-pot synthesis at 40 °C without the addition of citrate. Although no template was used during synthesis, spherical nanoparticles were obtained (Figures 1A and S1) with a size of about 119-241 nm as determined from SEM images (Table 1). The low synthesis temperature and alkaline pH used here have been reported to favor the formation of spherical nHA.^18,19^ However, the particles agglomerated quickly, as indicated by the large hydrodynamic size in the range of micrometers measured by DLS (Table 1), likely due to their neutral zeta potential of around 1 mV (Table 1).

**Table 1:** Characterization of nHA Synthesized with (+)/without (−) Citrate at 40 °C at Different Aging Times

	size_SEM_ (nm)	size_DLS_ (nm)	PDI	zeta (mV)	Ca/P ratio	crystallinity index
nHA–cit 10 min	156 ± 40	5321 ± 708	0.660 ± 0.024	0.6 ± 0.1	1.565 ± 0.005	n/a
nHA +cit 10 min	163 ± 54	4875 ± 387	0.345 ± 0.059	–0.6 ± 0.1	1.574 ± 0.004	n/a
nHA–cit 1 h	241 ± 69	5734 ± 1478	0.496 ± 0.102	2.8 ± 0.2	1.585 ± 0.003	0.098
nHA + cit 1 h	119 ± 14	1068 ± 212	0.302 ± 0.016	–10.4 ± 1.0	1.579 ± 0.003	0.069
nHA–cit 5 h	223 ± 73	3943 ± 193	0.554 ± 0.037	0.6 ± 0.3	1.601 ± 0.005	0.134
nHA + cit 5 h	223 ± 63	272 ± 8	0.155 ± 0.006	–15.5 ± 1.3	1.623 ± 0.002	0.083

To improve the colloidal stability of nHA, we used citrate addition in the synthesis process, which decreased the surface charge to between −10 and −20 mV, as confirmed by the evident shift of the zeta potential (Figure 1B). The negative charge introduced by citrate as a dispersant increased the colloidal stability of the particles (Figure 1C) and decreased their hydrodynamic size (Table 1). The effect of citrate addition on colloidal stability increased with aging time, as indicated by the strong decrease in particle size of citrate-modified nHA with increasing aging time. Most notably, no effect of citrate addition was observed for nHA aged for 10 min, likely because this short aging time is not sufficient to form stable nanoparticles without citrate addition. The smallest, most negatively charged nHA was obtained by synthesis using citrate as dispersant and an aging time of 5 h, resulting in particles with a hydrodynamic size of about 270 nm and a zeta potential of −16 mV. Of note, it should be emphasized that DLS is basically not reliable for quantification of micron-sized particles due to sedimentation of particles. However, the decrease in size upon citrate addition was also confirmed by confocal images of fluorescently labeled nHA (Figure S1B) and the lower polydispersity values (PDI) (Table 1). Longer aging times also increased the crystallinity of nHA, as evidenced by the sharper peaks in the XRD and FTIR graphs (Figure 1D) and the calculated crystallinity index (Table 1). FTIR measurements showed various vibrations of the phosphate groups, which became more narrow and defined with increasing aging time (Figure S1C), indicative of higher crystallinity.^20^ nHA synthesized using citrate as dispersant was slightly less crystalline than citrate-free nHA at similar aging times (Table 1), although the differences were minor. This minor difference in crystallinity did not significantly change nHA dissolution rate at pH 6 as evidenced by the absence of any statistical differences in the amount of calcium release over time for nHA aged for 5 h and synthesized with or without citrate as a dispersant (Figure 1E).

**Figure 2:**
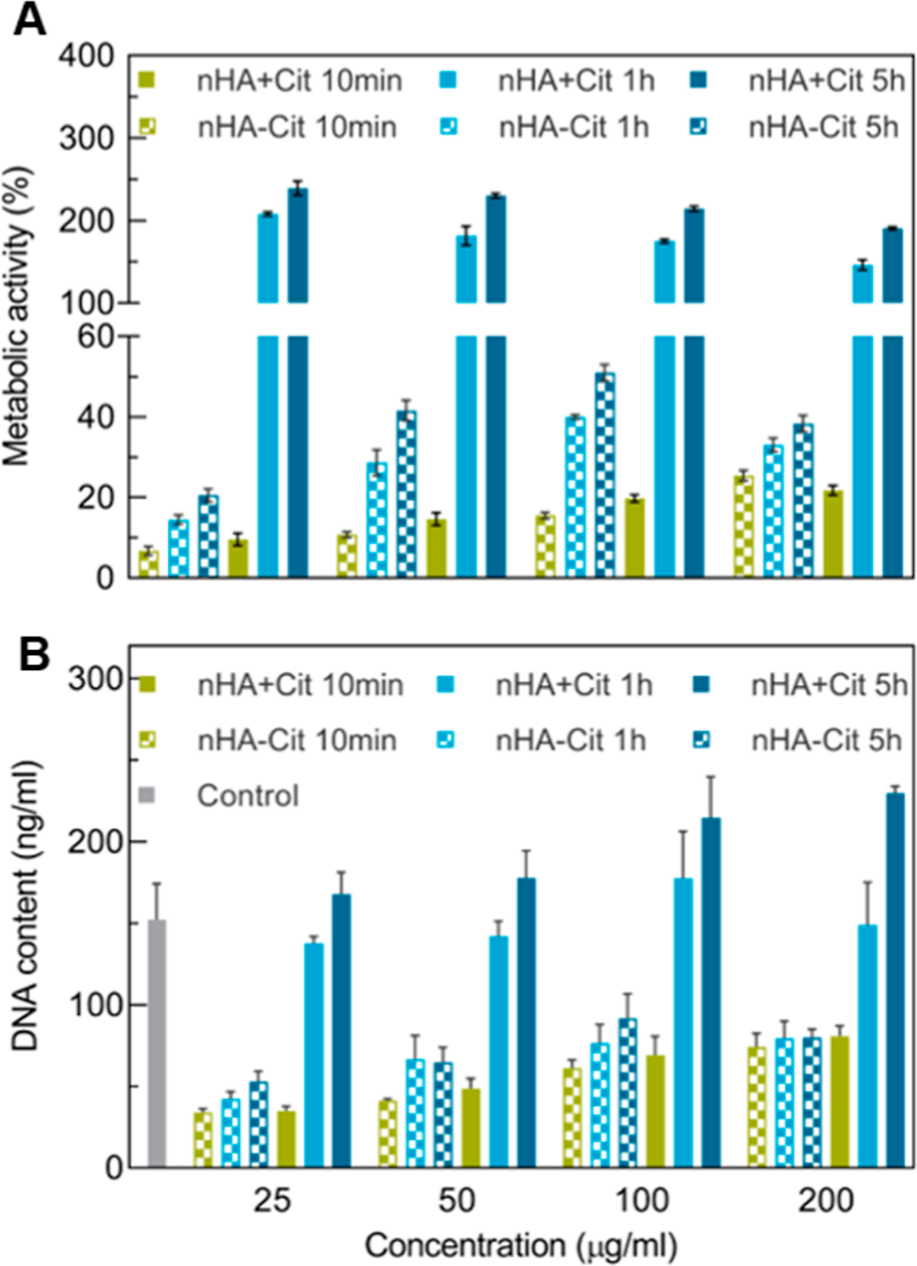
Cytocompatibility of nHA synthesized with (+)/without (−) citrate at 40 °C and aged for either 10 min, 1 or 5 h. (A) Metabolic activity and (B) DNA content of MC-3T3 cells after exposure to hydroxyapatite for 72 h. Untreated cells were set as 100%. Statistical analysis can be found in Table S1.

Next, we tested the cytocompatibility of these particles by measuring the metabolic activity of cells exposed to nHA with different aging times synthesized with/without citrate. After 24 h, all cells exposed to nHA showed metabolic activity similar to or higher than unexposed cells (Figure S2A), except nHA aged for 10 min, which showed lower metabolic activity of about 50–80% at concentrations of 25 and 50 μg/mL. Interestingly, an increase in nHA concentration corresponded with increased metabolic activity, suggesting either a larger number of living cells or elevated metabolic stress. After 72 h of exposure, striking differences were found between nHA synthesized with vs without citrate (Figure 2A). Cells exposed to agglomerated nHA without citrate were less metabolically active compared to the control (10–30%), suggesting cell death. This observation was further confirmed by DNA quantification, showing the low amounts of DNA present in those samples (Figure 2B). In contrast, cells exposed to colloidally stable nHA synthesized using citrate as dispersant showed increased metabolic activity compared to 24 h, which was about 2-fold higher than the control. An exception was nHA aged for 10 min (with citrate), which was comparable to nHA without citrate. This observation is consistent with the comparable size and zeta potential measured for these two particles (Table 1). Interestingly, for both nHA with and without citrate, metabolic activity increased with increasing aging time, which might be related to the slightly more crystalline structure of longer-aged nHA. Overall, these data show that citrate addition renders nHA negatively charged, thereby improving the colloidal stability through repulsive forces between the nanoparticles. nHA crystallinity can be varied by changing the aging time and/or addition of citrate. Cytocompatibility testing indicates that agglomeration seems to be the most dominant factor leading to cell death, while crystallinity affects nHA cytotoxicity to a lesser extent.

### Characterization and Cytocompatibility of Lyophilized nHA Synthesized at Different Temperatures

Generally, it is experimentally challenging to decouple the effects of separate nanoparticle parameters on cellular behavior since these parameters are often interrelated. Here, the addition of citrate to nHA not only improved its colloidal stability but also changed the surface charge and slightly reduced the crystallinity of nHA. However, we aimed to decouple these parameters to assess their separate effects on nHA cytocompatibility. To this end, we prepared new batches of citrate-modified nHA aged for 5 h since these particles exhibited the highest colloidal stability. Instead of studying aging time and citrate addition, we now tuned nHA crystallinity by performing nanoparticle synthesis at different temperatures (i.e., at 40 or 60 °C) since such variation is known to affect nHA crystallinity,^2,5^ whereas agglomeration was induced independently from surface charge and crystallinity by means of subsequent lyophilization. This synthesis yielded spherical nanoparticles (Figure S3A) with a size of 170–223 nm as determined from SEM images (Table 2), irrespective of the synthesis temperature. The surface charge of nHA prepared at 40 °C using citrate addition differed slightly from comparable particles reported above (Table 1), which we attribute to batch-to-batch variability. Nanoparticle crystallinity increased with an increasing synthesis temperature, as evidenced by more narrow diffraction peaks and a higher crystallinity index for particles prepared at 60 °C (Figure S3B and Table 2). Again, significant differences in nHA dissolution at pH 6 were not observed based on quantification of calcium release (Figure S3C). Lyophilization turned the dispersed nanoparticles into micrometer-sized agglomerates, as evidenced by DLS analysis (Table 2) and confocal microscopy (Figure S3D), and reduced the negative surface charge of the particles. However, it should be realized that assessment of zeta potential using electrophoretic light scattering was less reliable for lyophilized micron-sized agglomerates. Cytocompatibility testing showed a striking effect of lyophilization-induced particle agglomeration on cells exposed to nHA for 24 and 72 h (Figure S3E,F). The metabolic activity of cells exposed to lyophilized (agglomerated) nHA was about 2-fold lower after 24 h and up to 15-fold lower after 72 h as compared to cells exposed to nonlyophilized (colloidally stable) nHA. The agglomerated nHA (2–5 μm) were considerably smaller than the size of MC-3T3 cells (typically 20–50 μm).^21^ Consequently, we assume that the suffocation of these cells resulting from reduced oxygen and nutrient exchange due to full coverage of the cells by nHA is unlikely. Regarding the effect of crystallinity on nHA cytotoxicity, no significant differences were found between nHA synthesized at different temperatures. This lack of any effect of nanoparticle crystallinity on cell viability was most likely caused by the fact that the crystallinity of the tested particles aged for 5 h was considerably higher than the particles aged for shortened time periods, irrespective of the synthesis temperature. Consequently, we assume that nanoparticle crystallinity does not affect nHA cytotoxicity beyond a certain minimal crystallinity. Overall, we conclude that nHA agglomeration exerted a much stronger effect on nHA cytotoxicity than did nHA crystallinity.

**Table 2:** Characterization of nHA Synthesized with Citrate at 40 °C or 60 °C and Aged for 5 h

	size_SEM_ (nm)	size_DLS_ (nm)	PDI	zeta (mV)	Ca/P ratio	crystallinity index
nHA + cit 40 °C	223 ± 48	113 ± 2	0.159 ± 0.020	–23.4 ± 0.8	1.623 ± 0.002	0.175
nHA + cit 40 °C lyo	n/a	5547 ± 631	0.990 ± 0.017	–11.2 ± 0.4	1.623 ± 0.002	0.175
nHA + cit 60 °C	170 ± 31	180 ± 2	0.164 ± 0.016	–21.2 ± 1.9	1.675 ± 0.008	0.263
nHA + cit 60 °C lyo	n/a	1786 ± 306	1.00 ± 0	–10.7 ± 1.1	1.675 ± 0.008	0.263

Altogether, these data indicate that although both crystallinity and agglomeration influence nHA cytotoxicity, agglomeration exerts the most pronounced effect on the cytocompatibility of nHA. The addition of citrate only improved the cytocompatibility of nHA aged for 1 and 5 h but not for nHA aged for 10 min. Considering the more pronounced effect of agglomeration compared to crystallinity, we investigated the effect of agglomeration on nHA cytocompatibility in more detail by means of nanoparticle internalization and intracellular calcium characterization by comparing two selected data sets, i.e. (i) nHA synthesized at 40 °C aged for 5 h with and without citrate (hereafter referred to as nHA–Cit and nHA + Cit, respectively) and (ii) nHA synthesized with citrate at 60 °C and aged for 5 h with or without lyophilization (referred to as nHA–lyo and nHA + lyo).

### Internalization of Hydroxyapatite Nanoparticles

**Figure 3:**
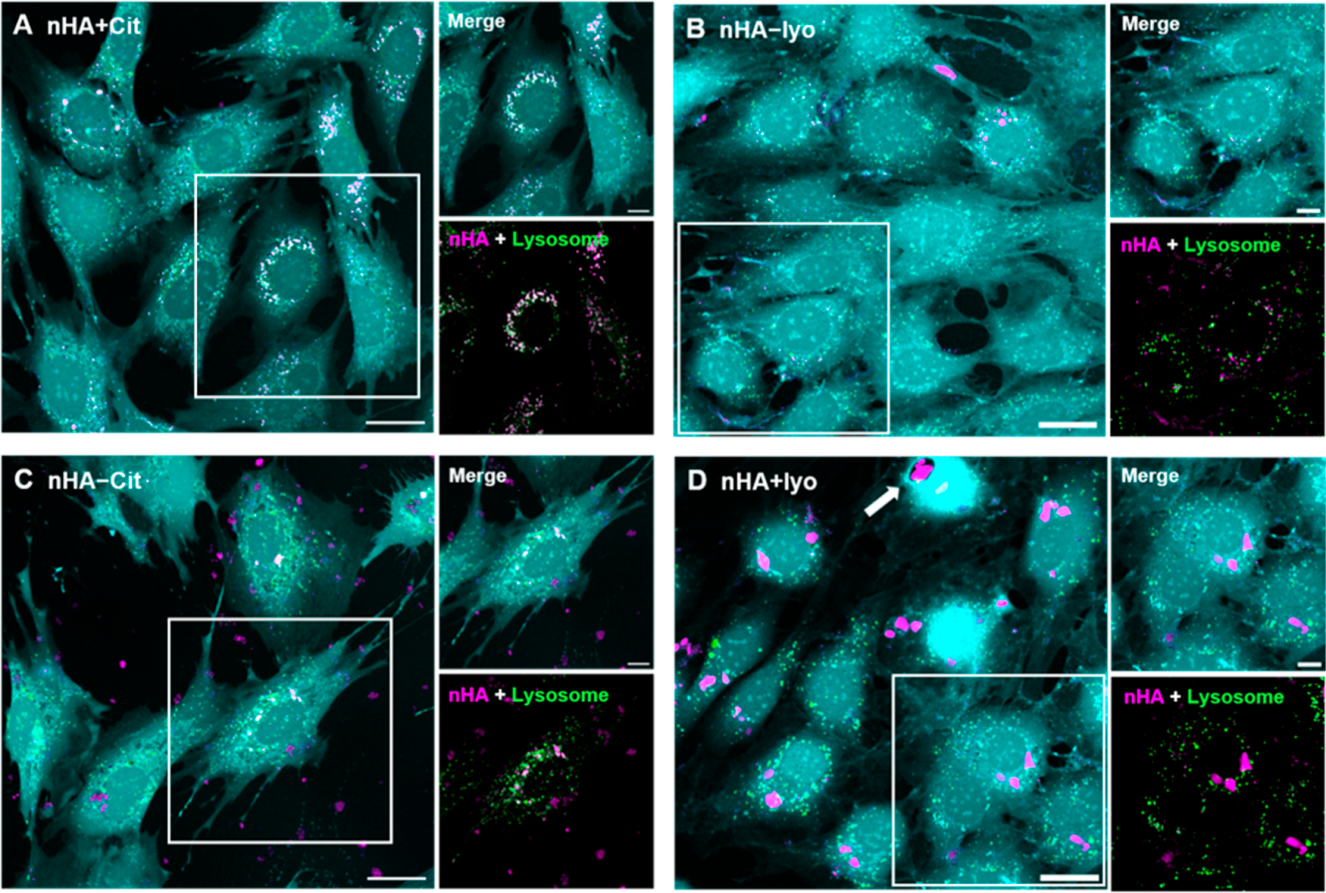
Internalization of nHA. Confocal live cell images showing internalization of nHA (green) after 24 h and their localization in lysosomal compartments (magenta). Co-localization of nHA and lysosomal compartments appears as white. (A/C) nHA were synthesized at 40 °C with/without citrate, or (B/D) synthesized at 60 °C with citrate and agglomerated by lyophilization. All nanoparticles were aged for 5 h. Enlarged regions of interest are marked by white squares in the images on the left, and a vacuole-like structure is indicated by the white arrow. Scale bars represent 25 and 10 μm (zoom-in image).

In an attempt to unravel the mechanism by which nanoparticle agglomeration enhances toxicity, we first investigated the cellular uptake and intracellular localization of the above-selected nHA. Fluorescently labeled nHA displayed a zeta potential of about −19.5 mV, which was not significantly different from unlabeled nHA. After 24 h of incubation, abundant cellular uptake was clearly observed for all types of nHA (Figure 3). Colloidally stable nHA (nHA + Cit and nHA–lyo) appeared as small dots, which colocalized with lysosomal staining (depicted as white dots in the overlay), confirming uptake of nHA into lysosomal compartments (Figure 3A,B). Both agglomerated forms of nHA were larger than colloidally stable forms (Figure 3C,D), while nHA agglomerated by lyophilization was particularly clustered into much larger aggregates compared to nHA agglomerated due to a lack of citrate. These big clusters did not colocalize with lysosomal compartments, but instead vacuole-like structures were occasionally observed (Figure 3D, white arrow). In contrast, nHA without citrate formed smaller agglomerates, which abundantly colocalized with lysosomal compartments (depicted as white in the overlay, Figure 3C). These results are in agreement with previous studies reporting size-dependent vacuolization upon nanoparticle internalization.^22−24^ For both agglomerated forms of nHA, disturbed lysosomal structures were observed (larger and blurred green dots, Figure 3C,D). Overall, cellular uptake was confirmed for all types of nHA and led to lysosomal uptake for all types of nHA except for the most agglomerated lyophilized nHA. Lysosomal localization of nHA is a desirable feature for intracellular delivery as the acidic pH in the lysosome will dissolve nHA, which facilitates pH-responsive cargo delivery. However, dissolution of nHA also results in the release of soluble calcium ions. Calcium is an important signaling molecule involved in many cellular pathways, including metabolism, proliferation, but also regulated cell death.^25,26^ Moreover, uptake of nHA in lysosomes has been reported to lead to lysosomal rupture due to an increase in osmotic pressure.^7^ Here, while some lysosomal structure seemed disturbed, disruption of lysosomal integrity was not observed, as lysosomes were abundantly present in all cells, which was most likely caused by the fact that nanoparticle concentrations in our study were lower than 140 μg/mL, which has been reported as a threshold above which lysosomal rupture occurs.^7^

### Hydroxyapatite Nanoparticle Dissolution and Intracellular Calcium Release

**Figure 4:**
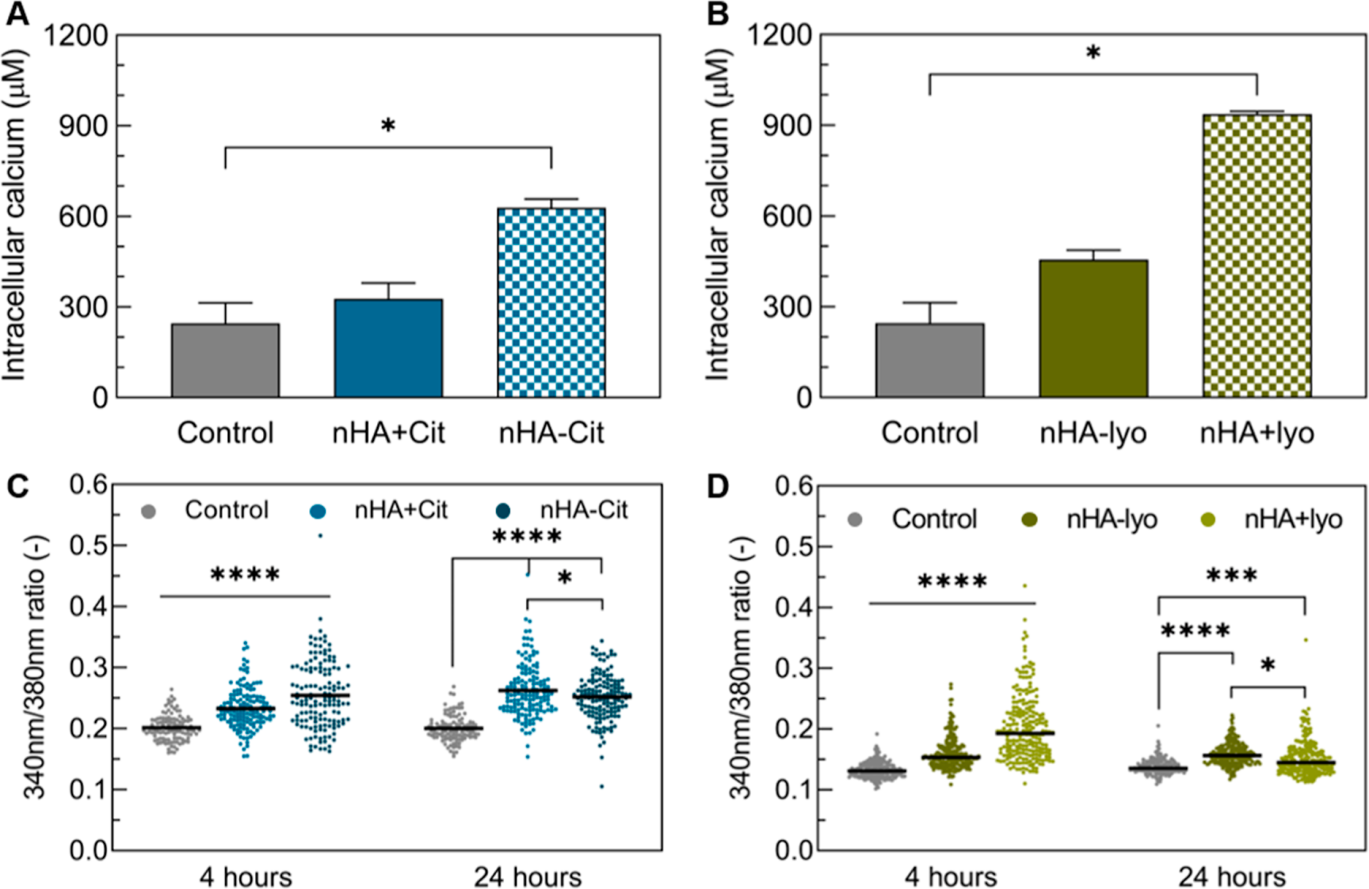
Intracellular calcium levels in MC-3T3 cells exposed to nHA (A/C) synthesized at 40 °C with/without citrate or (B/D) synthesized at 60 °C with citrate and agglomerated by lyophilization. All nanoparticles were aged for 5 h. Calcium levels were determined after 24 h using inductively coupled plasma mass spectrometry (A/B) or microscopically after 4 and 24 h using a calcium-sensitive dye (C/D).

In an attempt to explain nHA cytotoxicity based on intracellular calcium levels, we assessed whether nHA internalization led to disturbances in intracellular calcium levels since lysosomes are acidic compartments which can dissolve nHA at slightly acidic pH, i.e., below 6.5.^7^ As shown in Figures 1E and S3C, all nHA dissolved at pH 6 and released up to 4.5% of their total calcium content within 24 h. First, we measured intracellular calcium concentrations of cells exposed to nHA for 24 h using inductively coupled plasma mass spectrometry (ICP–MS). These cells showed higher levels of intracellular calcium compared to the untreated control (Figure 4A,B). Exposure to colloidally stable nHA (nHA + Cit and nHA–lyo) increased the intracellular calcium content by 1.6-fold and 1.5-fold, respectively, while agglomerated nHA (nHA–Cit and nHA + lyo) increased intracellular calcium by 3.1-fold compared to the control. Importantly, these results represent the maximal intracellular calcium concentration in the case that all internalized nHA dissolves in the lysosome. Due to the sample preparation for ICP–MS, which requires addition of nitric acid, potentially also nHA which was not yet dissolved in cells was measured, likely leading to overestimation of intracellular calcium concentration using ICP–MS. Therefore, we also assessed intracellular calcium levels by using calcium imaging. This method is based on the absorbance maximum shift of a fluorescent dye upon binding of dissolved calcium ions. The ratio between bound and unbound dyes is a measure for the intracellular calcium concentration. Generally, cells exposed to any type of nHA for 4 or 24 h showed higher calcium levels and a wider data distribution within the cell population compared to their experiment-matched control (Figure 4C,D). Strikingly, differences in basal calcium levels (control) were found between the two data sets, which may be due to batch differences between the cells. Of note, the data herein were acquired on a discrete time point as time lapse imaging was not feasible due to photobleaching of the dye. Overall, these results indicate that exposure to nHA can increase intracellular calcium levels. A previous study reported intracellular calcium fluctuations with spikes after exposure to unfunctionalized calcium phosphate nanoparticles.^17^ However, these spikes were observed in only about 40% of the cells, which would explain the wide data distribution observed herein. Interestingly, a recent study discovered calcium-rich multivesicular bodies in cells exposed to nHA and suggested these may act as calcium reservoirs.^27^ Although elevated intracellular calcium has previously been reported to correlate with cytotoxicity of nHA,^8^ it remains to be elucidated if the changes in intracellular calcium observed herein, which were relatively small compared to cells treated in a control experiment using an ionophore to stimulate calcium influx (Figure S4), are the main driver leading to the large differences in cytotoxicity observed (see Figures 2 and S3).

### Reactive Oxygen Species

**Figure 5:**
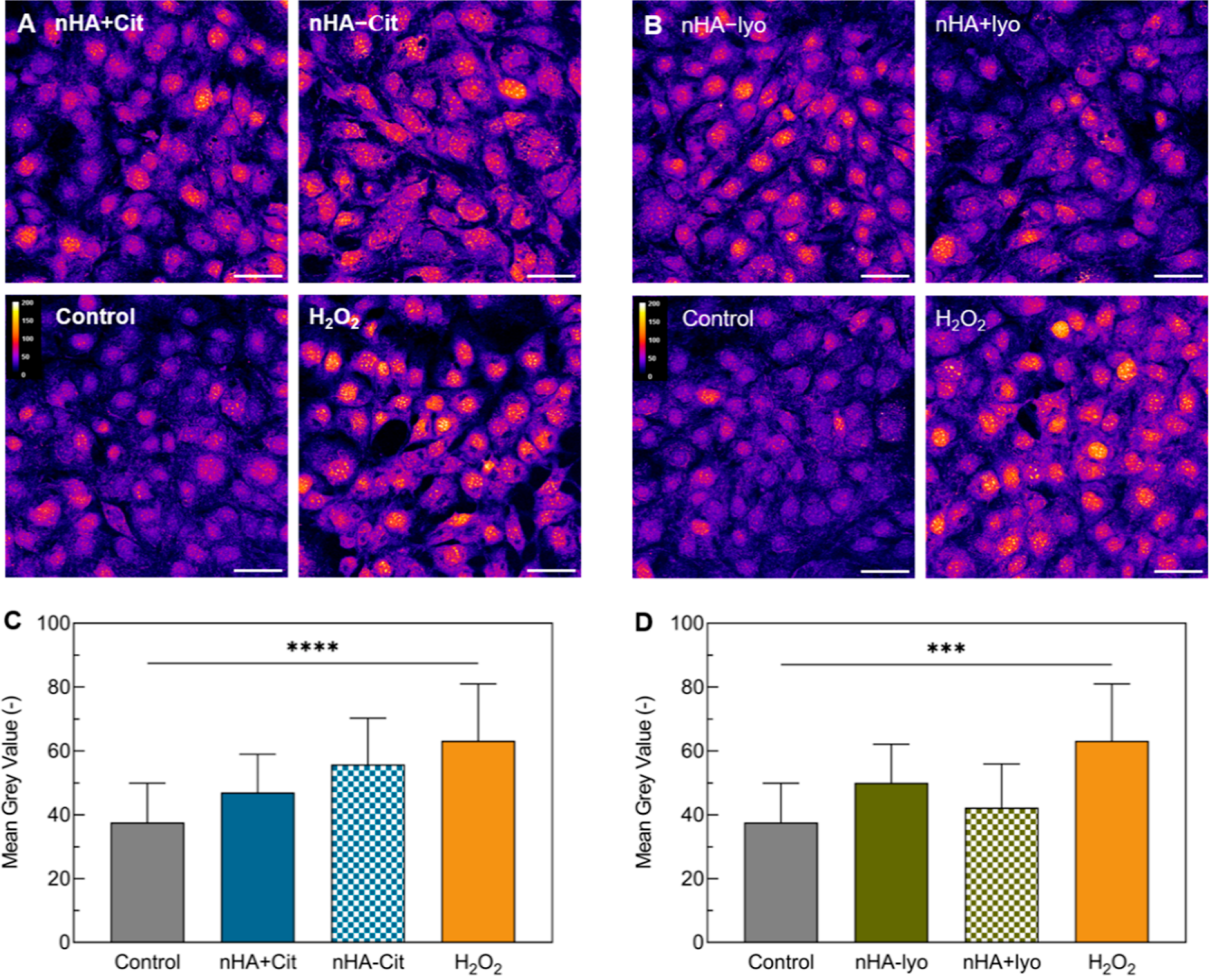
ROS after exposure to nHA. Confocal live cell images of MC-3T3 cells exposed to (A) nHA synthesized at 40 °C with/without citrate aged for 5 h and (B) nHA synthesized at 60 °C with citrate aged for 5 h and agglomerated by lyophilization. Cells were stained for ROS. Images are colored with Fire LUT to indicate ROS intensity. (C,D) Quantification of staining intensity. Scale bar represents 50 μm.

The production of reactive oxygen species (ROS) is often proposed as a mechanism behind cytotoxicity of nanoparticles.^12,13^ We therefore assessed ROS levels in cells exposed to colloidally stable and agglomerated nHA for 24 h using CellROX green staining, a dye that becomes fluorescent upon oxidation.^28^ Cells exposed to nHA showed increased ROS staining compared to the untreated control, as evidenced by the lighter color in the images (Figure 5A/B). Agglomerated nHA without citrate (nHA–Cit) led to more intense staining compared with colloidally stable nHA (nHA + Cit). By quantifying the average nuclear signal intensity of ROS staining, colloidally stable nHA showed 1.3-fold higher signal compared to the control, and agglomerated nHA showed 1.5-fold and 1.2-fold higher signal intensity compared to the control and colloidally stable nHA, respectively (Figure 5C). In contrast, for nHA with similar crystallinity (nHA–lyo and nHA + lyo), the ROS signal was higher for colloidally stable nHA compared to agglomerated nHA (nHA + lyo), as confirmed by the lighter color in the images (Figure 5B) and the 1.2-fold higher signal intensity of cells exposed to colloidally stable nHA than cells exposed to agglomerated nHA (Figure 5D). We speculate that the lower ROS production observed for nHA agglomerated by lyophilization (nHA + lyo) may be related to its intracellular location. As shown in Figure 3, unlike agglomerated nHA without citrate, no clear colocalization of nHA + lyo and lysosomes was observed. Moreover, nHA + lyo was occasionally found in vacuole-like structures. Overall, these results confirm previous reports that exposure to nHA increases ROS production.^14,29^ However, whether increased ROS production is linked to elevated intracellular calcium, as suggested by others,^10,25^ cannot be concluded from the results presented herein.

By combining all evidence presented herein, we conclude that agglomeration renders nHA more cytotoxic, while nHA-induced cell death is likely linked to several mechanisms. On the one hand, elevated intracellular calcium and ROS production have been observed for smaller agglomerates of nHA (nHA–Cit). On the other hand, large agglomerates (nHA + lyo) increased intracellular calcium and ROS only marginally but reduced metabolic activity drastically (up to 15-fold) after 72 h of exposure. For these larger agglomerates, localization in vacuole-like structures was observed, a phenomenon previously reported for nHA and other nanoparticles,^22−24^ potentially indicating autophagy. Autophagy, cellular “self-digestion”, can act as a self-defense mechanism to eliminate nanoparticles and contributes to programmed cell death.^30,31^ Thus, the vacuolization observed could be a sign of autophagy. To prove this hypothesis, further investigation into autophagic markers and pathways is required, especially because the vacuolization pathway has been shown to be independent from autophagy, although nanoparticles were shown to induce both vacuolization and autophagy.^23^

## Conclusions

nHA are promising nanocarriers to facilitate intracellular drug delivery due to their high binding affinity and pH-dependent solubility. Yet, the dependence of nHA cytocompatibility on their physicochemical properties remains unclear since numerous studies have revealed starkly contrasting results. Herein, we show that both low crystallinity and, more significantly, agglomeration strongly enhances nHA cytotoxicity. Such cytotoxic effects could be linked to elevated intracellular calcium levels, increased ROS production, and/or activation of the autophagic pathway. These findings provide important guidelines for the future design of nHA-containing formulations, indicating that nHA crystallinity and especially agglomeration should be carefully controlled depending on the intended biomedical application.

## Experimental Section

### Synthesis of Hydroxyapatite Nanoparticles

nHA were synthesized by a one-pot wet-chemical synthesis at either 40 or 60 °C. An aqueous solution of 100 mM sodium phosphate tribasic dodecahydrate (Sigma-Aldrich) was heated to either 40 or 60 °C under magnetic stirring. When the required synthesis temperature was reached, an equal amount of 167 mM calcium acetate monohydrate (Sigma-Aldrich) was added while being stirred vigorously at 1000 rpm. The solution was covered and left to stir at 500 rpm for 2 h. Afterward, 1.88 g of trisodium citrate dihydrate (Merck) was added as dispersant, and the reaction was left to stir for another 3 h. The nanoparticles were then collected by centrifugation at 16,800 rcf for 10 min. The resulting pellet was washed twice with demineralized water by resuspension through sonication (10 min) and vortexing. nHA was then resuspended in demineralized water. Half of the suspension was lyophilized and stored dry at room temperature, while the other half of the suspension was stored at 4 °C.

### Characterization of Hydroxyapatite Nanoparticles

The hydrodynamic size of nHA was measured by dynamic light scattering (DLS) in demineralized water using a Malvern Zetasizer Lab (Malvern PANalytical), and their surface charge was determined by zeta potential measurement in 5 mM HEPES buffer (Merck) at pH 7.4. nHA morphology was assessed by scanning electron microscopy (SEM). For SEM sample preparation, nHA was diluted 1000 times in demineralized water, and 10 μL was dropped on a copper grid and allowed to air-dry. Samples were then sputter-coated with gold and imaged with a Zeiss Sigma 300 field-emission scanning electron microscope. The average size of nHA in its dry state was determined by measuring 50 nanoparticles from SEM images using open-source Fiji software. The concentration of nHA was determined by lyophilizing 0.5 mL of nHA suspension and weighing the resultant dried powder (*n* = 2). Structural characterization of nHA was conducted by using powder X-ray diffraction (XRD) and attenuated total reflection Fourier transform-infrared spectroscopy (ATR-FTIR) on the lyophilized samples. XRD patterns were produced using an X-ray diffractometer (XRD X’Pert3 Powder, Malvern PANalytical) with Cu Kα radiation (λ = 1.154) generated at 45 kV and 40 mA. Samples were scanned in the 2θ range from 20 to 55°, with a step size of 0.01° and a step time of 3 s. The crystallinity index (CI) was calculated based on the (002) reflections represented in the 26° peak using the formula
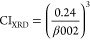
in which β002 represents the full width at half-maximum (fwhm) of (002) reflections.^32^ ATR-FTIR (Spectrum Two FT-IR Spectrometer, PerkinElmer) spectroscopy was used to identify the molecular groups present in the synthesized nHA. The spectrum was recorded in the range 400–4000 cm^–1^ with a resolution of 4 cm^–1^.

### Calcium Release upon nHA Dissolution

The dissolution rate of nHA was measured by quantifying calcium release over time at pH 6.0 by using inductively coupled plasma-optical emission spectroscopy (ICP–OES). Since lysosomal pH is reported at around pH 4.5,^7^ we selected a slightly higher pH to test nHA dissolution to prevent overestimation of intracellular calcium release. 10 mg of lyophilized nHA was immersed in 1 mL of 0.1 M HEPES buffer (pH 6.0) in Eppendorf tubes. Tubes were incubated at 37 °C under static conditions. At predefined time points, the samples were centrifuged at 9391 rcf for 10 min, and 0.8 mL of the supernatant was collected. The same volume of fresh buffer was added for further dissolution. For analysis by means of ICP–OES (iCAP 6000 series, Thermo scientific), 0.2 mL of the supernatant was diluted with 4.8 mL of demineralized water and mixed with 5 mL of 2% nitric acid (Merck). To determine the calcium-to-phosphate ratio (Ca/P), 10 mg of lyophilized powder was dissolved in 1 mL of 10% nitric acid (Merck), diluted 10× with demineralized water, and further diluted 50x in 1% nitric acid for analysis by ICP–OES.

### Cell Culture

The murine preosteoblast cell line MC3T3-E1 subclone 4 (CRL-2593, American Type Culture Collection) was maintained in Minimal Essential Medium α (Gibco, MEM-α without ascorbic acid), supplemented with 10% FBS and 100 units/mL penicillin and 0.1 mg/mL streptomycin (Sigma-Aldrich). These cells were selected in view of their potential for osteogenic differentiation.

### Cytotoxicity of nHA

#### Metabolic Activity

To assess cytotoxicity of nHA, the metabolic activity of cells was measured by Cell Counting Kit 8 (CCK-8) after culture with different concentrations of nHA for 24, 48, and 72 h. Cells were seeded at 7500 cells/cm^2^ and left to adhere overnight. A serial dilution of nHA (200, 100, 50, and 25 μg/mL) was prepared in medium and added to the cells. A stock solution of 5 mM water-soluble tetrazolium 8 (WST-8, 5-(2,4-disulfophenyl)-3-(2-methoxy-4-nitrophenyl)-2-(4-nitrophenyl)-2H-tetrazolium, inner salt, monosodium salt, Cayman Chemicals) and 0.2 mM 1-methoxy-5-methylphenazinium (TCI Chemicals) was prepared in 150 mM sodium chloride (Merck) and stored at −80 °C until further use. At the respective time point, the cells were washed thrice with DPBS (Gibco) and a 10 v/v % working solution of CCK-8 in full medium was added. After 4 h of incubation, 100 μL of supernatant was transferred to a new well plate and absorbance was read at 460 and 650 nm (reference). The remaining CCK-8 solution was removed, and cells were washed three times with DPBS before lysis with 100 μL of cell lysis buffer (1 mM tris (Invitrogen), 5 mM sodium chloride (Merck), and 0.5 mM EDTA (Sigma-Aldrich) in demineralized water and sterile filtered through a 0.2 μm syringe filter (Merck)) for subsequent DNA assay.

#### DNA Assay

DNA content was quantified as a measure for cell proliferation using the QuantiFluor dsDNA kit (Promega) according to the manufacturer’s instructions. In brief, 50 μL of cell lysate was mixed with 50 μL of QuantFluor dye solution (1:200 in Tris–EDTA buffer) and left to react for 5 min before reading the fluorescence at excitation 504 nm and emission 531 nm. Absolute DNA concentrations were calculated based on a standard curve prepared with lambda standard DNA (part of the kit).

### Cellular Uptake of Hydroxyapatite Nanoparticles

#### Fluorescent Labeling of Hydroxyapatite Nanoparticles

nHA were labeled using AlexaFluor-647-risedronate (AF647-RIS, Biovinc), a fluorescent bisphosphonate dye that binds to calcium phosphate. nHA were diluted to 1 mg/mL in demineralized water. Fluorescent dye (0.01 nM in DPBS) was added to the nHA suspension in a ratio of 1:10, mixed well, and left to react for 10 min. The fluorescently labeled nHA was used without further washing. To correct for the uptake of unreacted dye, a control containing the same dilution of dye in demineralized water without nHA was prepared.

#### Visualization of Cellular Uptake of Hydroxyapatite Nanoparticles

10,000 cells/cm^2^ were seeded in an 8-well slide (ibidi) and left to adhere overnight. The next day, cells were stained with 1 μM CellTrace yellow (Invitrogen) in DPBS according to the manufacturer’s instructions. Then, AF647-RIS-labeled nHA were diluted in full medium to 50 μg/mL and added to the cells. The same volume of labeling solution as used in the dilution of nHA was used as a control to later correct for signal originating from uptake of free fluorescent dye. After 24 h, the nHA-containing medium was removed, and cells were washed twice with DPBS and lysosomal compartments were stained with 50 nM LysoTracker green (ThermoFisher Scientific) in HEPES-Tris buffer (132 mM sodium chloride (Merck), 5.5 mM D-(+)-glucose (Merck), 10 mM Hepes (Sigma-Aldrich), 4.2 mM potassium chloride (Merck), 1 mM magnesium chloride (Merck), 1 mM calcium chloride (Merck) in demineralized water, pH adjusted to 7.4 using 1 M Tris (Invitrogen) and sterile filtered) 30 min prior to imaging. The internalization of nHA was visualized using a Leica TCS SP8 SMD confocal microscope (Leica Microsystems), equipped with an HCX PL APO 63*x*/0.40 water immersion objective and a temperature-controlled stage at 36.5 °C. Fluorophores were excited using a white-light laser, and emissions were detected with hybrid detectors (HyD). LysoTracker green was excited at 488 nm (detection: 500–540 nm); CellTrace yellow was excited at 561 nm (detection: 580–620 nm), and AF647-RIS-nHA was excited at 633 nm (detection: 650–700 nm). Fiji was used for the reconstruction of the images.

#### Intracellular Calcium

Changes in intracellular calcium levels after exposure to nHA were assessed both optically and analytically. Imaging with calcium-sensitive dyes was used to obtain qualitative information about relative calcium levels between groups and visual information about the state and morphology of the cells, while mass spectrometry was used to obtain quantitative information on intracellular calcium concentrations.

#### Intracellular Calcium Measurement with ICP–MS

Cells were seeded at 3500 cell/cm^2^ in T175 flasks and left to grow until they reached 70% confluency (2–3 days). When 70% confluency was reached, nHA diluted to 50 μg/mL in full medium was added. After 24 h of incubation, cells were washed four times with DPBS to remove extracellular calcium and subsequently detached using trypsin-EDTA. Trypsinization was performed twice for cells exposed to nHA, as detachment was less efficient compared to unexposed control cells. Cells were collected and washed twice with DPBS by centrifugation (300 rcf, 5 min) before counting. The cells were then collected, resuspended in 1.5 mL of Milli-Q water, and freeze–thawed thrice to lyse the cells. The cell lysate was filtered through a 0.2 μm syringe filter before diluting 1:1 with 4% nitric acid for ICP–MS measurement.

#### Calcium Imaging with Fura-2 AM

10,000 cells/cm^2^ were seeded in a 35 mm dish (ibidi) and left to adhere overnight. The next day, nHA was diluted in the full medium to 50 μg/mL and added to the cells. After 4 or 24 h, the cells were washed three times with DPBS and stained with 3 μM Fura-2 AM (Invitrogen) in HEPES-Tris buffer for 30 min at room temperature protected from light. Thereafter, the staining solution was removed, and cells were incubated for another 20 min in HEPES-Tris buffer at room temperature. Before imaging, HEPES-Tris buffer was removed and refreshed with 0.5 mL. Samples were then placed in the stage incubator and left to equilibrate for 5 min at 37 °C. Untreated cells were used as a control. As a positive control, cells were treated with 1 μM ionomycin (ionomycin calcium salt, Sigma, Cat no. I0634) during imaging to stimulate calcium influx, and calcium dynamics were captured during 10 min at a frequency of one image/second. Images were acquired with a Zeiss Axio Observer 7 inverted fluorescent microscope equipped with a Fluar 40*x*/1.30 oil immersion objective, fast filter wheels, and a stage incubator (ibidi) set at 37 °C and 5% CO_2_ under a humidified atmosphere. Fura-2 was excited with a Sutter Lambda DG5 light source at 340 and 380 nm, and emission was recorded at 507–543 nm with an AxioCam702 with exposure time set to 20 ms. Fiji was used to determine signal intensity in the two channels to calculate the ratio of calcium-bound-to-unbound dye in the nucleus.

### ROS Staining

20,000 cells/cm^2^ were seeded in an 8-well slide (ibidi) and left to adhere overnight. The next day, AF647-RIS-nHA were diluted in full medium to 50 μg/mL and added to the cells. After 24 h, the nHA-containing medium was removed and cells were washed thrice with DPBS. CellROX green reagent (Invitrogen) was diluted to 5 μM and Hoechst (Invitrogen) was diluted to 2.5 μg/mL in full cell culture medium. Cells were incubated with the staining solution for 30 min at 37 °C. Then, the staining solution was removed and cells were washed once with DPBS before adding HEPES-Tris buffer for imaging. As a positive control, cells were incubated with 50 μM hydrogen peroxide (Merck) in DPBS for 30 min before staining with CellROX green reagent. Images were acquired with a Leica TCS SP8 SMD confocal microscope equipped with an HC PL APO CS 40*x*/0.85 air objective and a temperature-controlled stage at 36.5 °C. Fluorophores were excited using a 405 diode and a white-light laser, and emissions were detected with a photo multiplier tube (PMT) and hybrid detectors (HyD). Hoechst was excited at 405 nm (detection: 420–480 nm); CellRox green reagent was excited at 488 nm (detection: 500–570 nm); and AF647-RIS-nHA was excited at 633 nm (detection: 650–700 nm). Fiji was used for the reconstruction and quantification of images.

### Statistical Analysis

Prism version 8.4 (GraphPad) was used for statistical analysis. Outliers were identified by robust regression and outlier removal (ROUT method) with *Q* = 5% and removed prior to statistical analysis. Calcium release data were analyzed by multiple *t*-test to compare the different nHA per time point. Release experiments were performed in triplicate (*n* = 3). Cell metabolic activity and calcium imaging data were analyzed by two-way analysis of variance (ANOVA) with Tukey multiple comparison correction to detect differences between the different nHA groups. Cytocompatibility experiments were performed in triplicate (*n* = 3) and repeated three times. For calcium imaging, 150–225 cells of two experiments performed on independent days were analyzed. Intracellular calcium data obtained using ICP–MS were analyzed by a Kruskal–Wallis test with Dunn’s multiple comparison correction. Experiments were performed in duplicate or triplicate (*n* = 2–3) and controls were averaged over two experiments. Signal intensity of ROS staining was measured in 270–360 nuclei and analyzed by one-way ANOVA with Tukey’s multiple comparison correction. All data are presented as mean ± standard deviation. Significance was set at *p* < 0.05 and *p* values are reported using *p < 0.05, **p < 0.01, ***p < 0.001, and ****p < 0.0001.

## Supporting Information Available

The following files are available free of charge. Characterization of nHA; cytocompatibility of nHA synthesized with/without citrate at 40 °C; statistical analysis of metabolic activity shown in Figures 2 and S2; characterization and cytocompatibility of citrate-modified nHA synthesized at 40 or 60 °C and aged for 5 h prior to agglomeration induced by lyophilization; statistical analysis of metabolic activity shown in Figure S3; and dynamics of intracellular calcium assessed microscopically using a calcium-sensitive dye in MC-3T3 cells treated with ionomycin to stimulate calcium influx (PDF)
