# Supplementary material for: Effect of Hydroxyapatite Nanoparticle Crystallinity and Colloidal Stability on Cytotoxicity
Source: ACS Biomater Sci Eng. 2024 Oct 7;10(11):6964–73. doi: 10.1021/acsbiomaterials.4c01283 (PMC11558557; doi:10.1021/acsbiomaterials.4c01283)
Supplement: Supplementary file 1 — ab4c01283_si_002.pdf [file ab4c01283_si_002.pdf]

Supporting Information

The effect of hydroxyapatite nanoparticle crystallinity and colloidal stability on cytotoxicity

*Lea Andrée[1], Lucas S. Joziasse[1], Merel J. W. Adjobo-Hermans[2], Fang Yang[1], Rong Wang[1], Sander C. G. Leeuwenburgh[1]**

[1] Department of Dentistry – Regenerative Biomaterials, Radboud University Medical Center, Nijmegen, The Netherlands

[2] Department of Medical BioSciences, Radboud University Medical Center, Nijmegen, The Netherlands

* Corresponding author: sander.leeuwenburgh@radboudumc.nl

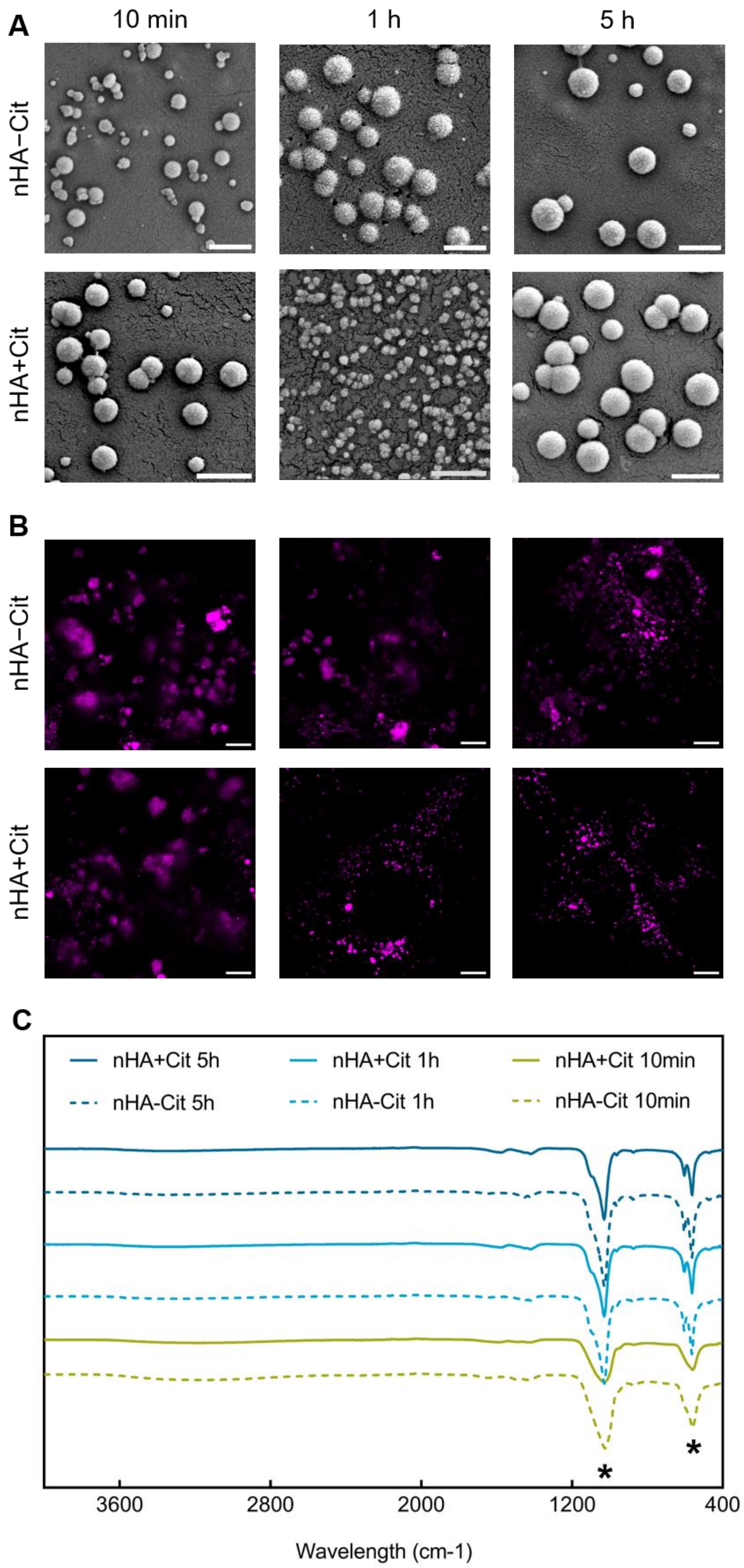

Figure S1. Characterization of hydroxyapatite nanoparticles. (A) Scanning electron microscopy images of nanoparticle morphology, (B) confocal images and (C) Fourier-transform infrared spectroscopy of hydroxyapatite nanoparticles synthesized at 40°C with (+) / without (-) citrate and different aging time. * indicates phosphate peaks. Scale bar represents 500 nm (A) and 10 µm (B) .

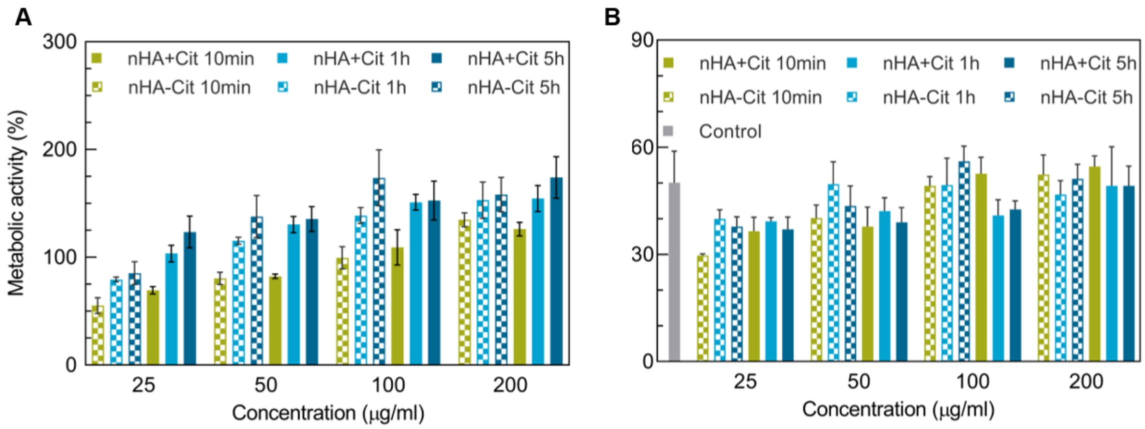

Figure S2. Cytocompatibility of hydroxyapatite nanoparticles synthesized with/without citrate at 40°C. (A) metabolic activity and (B) DNA content of MC-3T3 cells after exposure to hydroxyapatite for 24 hours. Untreated cells were set as 100%. Statistical analysis can be found in Table S1.

Table S1. Statistical analysis of metabolic activity shown in Figure 2 and Figure S2.

**24 hours**	**25 μg/mL**	**50 μg/mL**	**100 μg/mL**	**200 μg/mL**
nHA-Cit 5h vs nHA-Cit 1h	ns	ns	*	ns
nHA-Cit 5h vs nHA-Cit 10min	*	****	****	ns
nHA-Cit 1h vs nHA-Cit 10min	ns	*	**	ns
nHA+Cit 5h vs nHA+Cit 1h	ns	ns	ns	ns
nHA+Cit 5h vs nHA+Cit 10min	****	****	**	***
nHA+Cit 1h vs nHA+Cit 10min	*	***	**	ns
nHA-Cit 5h vs nHA+Cit 5h	**	ns	ns	ns
nHA-Cit 1h vs nHA+Cit 1h	ns	ns	ns	ns
nHA-Cit 10min vs nHA+Cit 10 min	ns	ns	ns	ns
**72 hours**				
nHA-Cit 5h vs nHA-Cit 1h	ns	***	**	ns
nHA-Cit 5h vs nHA-Cit 10min	***	****	****	***
nHA-Cit 1h vs nHA-Cit 10min	****	****	****	ns
nHA+Cit 5h vs nHA+Cit 1h	****	****	****	****
nHA+Cit 5h vs nHA+Cit 10min	****	****	****	****
nHA+Cit 1h vs nHA+Cit 10min	****	****	****	****
nHA-Cit 5h vs nHA+Cit 5h	****	****	****	****
nHA-Cit 1h vs nHA+Cit 1h	****	****	****	****
nHA-Cit 10min vs nHA+Cit 10 min	ns	ns	ns	ns

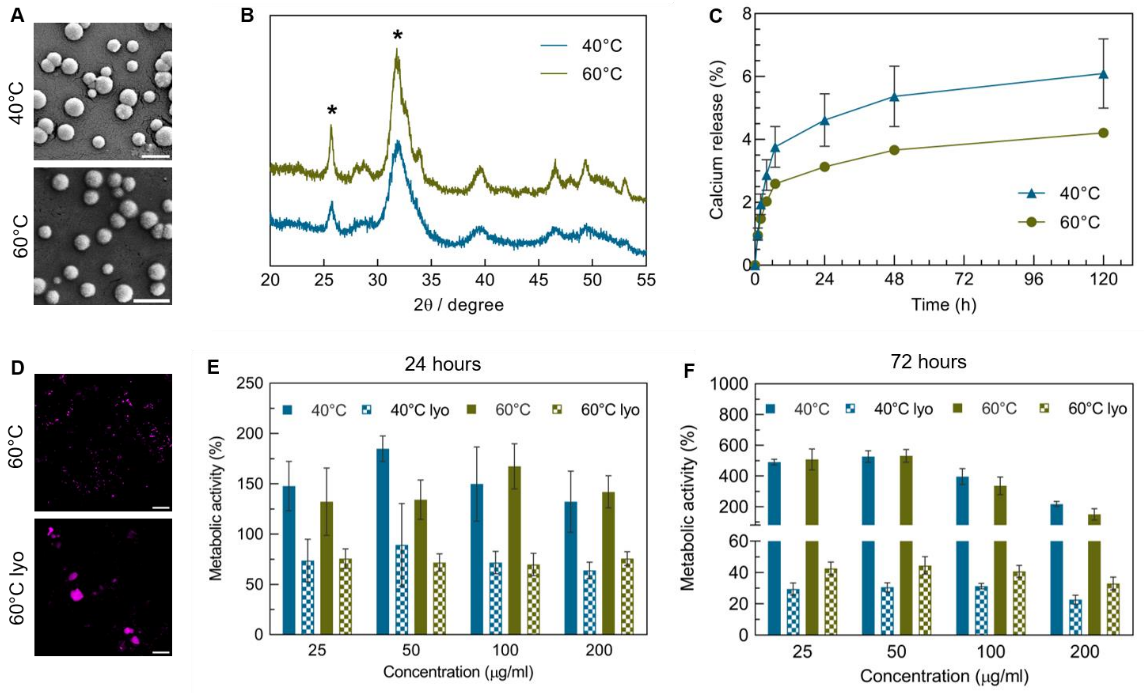

Figure S3. Characterization and cytocompatibility of citrate-modified hydroxyapatite nanoparticles synthesized at 40 °C or 60 °C and aged for 5 hours prior to agglomeration induced by lyophilization. (A) Scanning electron microscopy images, (B) X-ray diffractograms, (C) Calcium release at pH 6, (D) Confocal images of fluorescently labeled nHA, Metabolic activity of MC-3T3 cells after exposure to nanoparticles for (E) 24 hours and (F) 72 hours. Untreated cells were set as 100%. Corresponding statistical analysis can be found in Table S2. * indicates peaks characteristic for apatite. SD was too small to be plotted (C). Scale bar represents 500 nm (A) and 10 µm (B).

Table S2. Statistical analysis of metabolic activity shown in Figure S3.

**24 hours**	**25 µg/mL**	**50 µg/mL**	**100 µg/mL**	**200 µg/mL**
40°C vs 40°C lyo	****	****	****	****
60°C vs 60°C lyo	***	***	****	****
40°C vs 60°C	ns	**	ns	ns
40°C lyo vs 60°C lyo	ns	ns	ns	ns
**72 hours**				
40°C vs 40°C lyo	****	****	****	****
60°C vs 60°C lyo	****	****	****	****
40°C vs 60°C	ns	ns	*	**
40°C lyo vs 60°C lyo	ns	ns	ns	ns

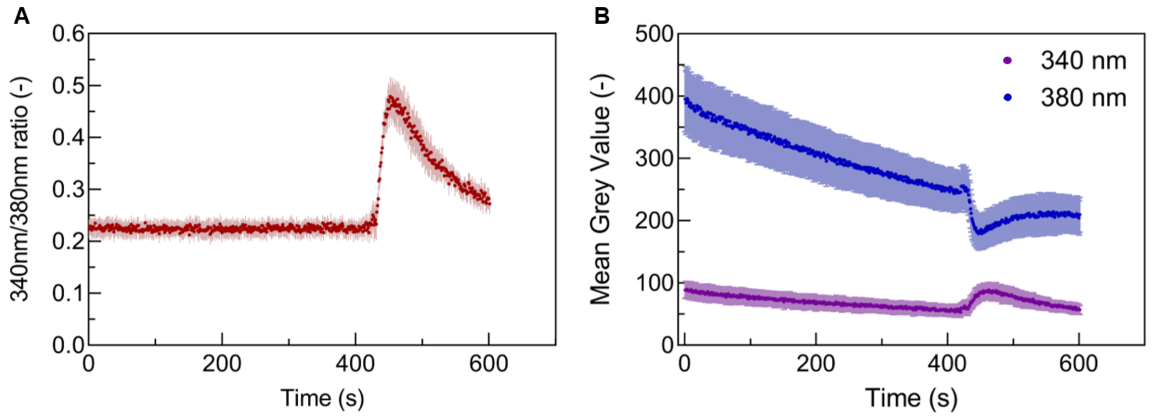

Figure S4. Dynamics of intracellular calcium assessed microscopically using a calcium-sensitive dye in MC-3T3 cells treated with ionomycin to stimulate calcium influx, showing (A) ratio between bound and unbound dye, and (B) signal intensity per channel (340 nm = Ca^{2+}-bound, 380 nm = Ca^{2+}-unbound).
